# Novel mutations in the *TBX5* gene in patients with Holt-Oram Syndrome

**DOI:** 10.1590/S1415-47572010005000051

**Published:** 2010-06-01

**Authors:** Marianna P. R. Porto, Naja Vergani, Antonio Carlos C. Carvalho, Mirlene C. S. P. Cernach, Decio Brunoni, Ana Beatriz A. Perez

**Affiliations:** 1 Department of Morfologia, Universidade Federal de São Paulo, São Paulo, SPBrazil; 2 Departamento de Medicina, Universidade Federal de São Paulo, São Paulo, SPBrazil

**Keywords:** Holt-Oram syndrome, congenital heart disease, *TBX5 gene*, *GATA gene*, *NKX2.5 gene*, mutation analysis

## Abstract

The Holt-Oram syndrome (HOS) is an autosomal dominant condition characterized by upper limb and cardiac malformations. Mutations in the *TBX5* gene cause HOS and have also been associated with isolated heart and arm defects. Interactions between the TBX5, GATA4 and NKX2.5 proteins have been reported in humans. We screened the *TBX5*, *GATA4*, and *NKX2.5* genes for mutations, by direct sequencing, in 32 unrelated patients presenting classical (8) or atypical HOS (1), isolated congenital heart defects (16) or isolated upper-limb malformations (7). Pathogenic mutations in the *TBX5* gene were found in four HOS patients, including two new mutations (c.374delG; c.678G > T) in typical patients, and the hotspot mutation c.835C > T in two patients, one of them with an atypical HOS phenotype involving lower-limb malformations. Two new mutations in the *GATA4* gene were found in association with isolated upper-limb malformations, but their clinical significance remains to be established. A previously described possibly pathogenic mutation in the *NKX2.5* gene (c.73C > 7) was detected in a patient with isolated heart malformations and also in his clinically normal father.

The Holt-Oram syndrome (HOS) (MIM #142900) is an autosomal dominant condition that has an estimated frequency of 1:100,000 live births (Basson *et al.*, 1997) and comprises malformation of the upper limbs (involving mainly the pre-axial ray) and congenital heart defects (CHD). Atypical phenotypes have also been described (Brassington *et al.*, 2003; Lehner *et al.*, 2003; McDermott *et al.*, 2005; Garavelli *et al.*, 2008) and may present as isolated skeletal anomalies or cardiac defects (Lehner *et al.*, 2003) or may include renal, craniofacial, axillary, tracheal and vertebral anomalies, deafness, abdominal *situs inversus* (Brassington *et al.*, 2003; McDermott *et al.*, 2005), and lower-limb malformations (Garavelli *et al.*, 2008). Due to the high variability of the phenotype, evaluation of the apparently asymptomatic relatives is highly recommended and should include anamnesis, upper-limb radiographs, and cardiac evaluation by electrocardiogram (ECG) and echocardiogram (ECO).

HOS is caused by mutations in the *TBX5* gene (Li *et al.*, 1997; Basson *et al.*, 1999). The *TBX5* gene (MIM *601620) maps to cytoband 12q24.1, belongs to the T-box family of transcription factors (Basson *et al.*, 1997; Li *et al.*, 1997) and is mainly expressed in the embryonic heart and upper-limb tissues (Bruneau *et al.*, 2001). Although the *TBX5* gene is responsible for most cases of HOS, the genetic heterogeneity of the syndrome can lead to false diagnosis, causing the mutation rate in sporadic and atypical cases to be underestimated (Mori and Bruneau, 2004).

The *GATA4* gene (MIM *600576) is a member of the GATA family of transcription factors, with expression mainly restricted to the heart and gonad tissues (Charron and Nemer, 1999). Protein GATA4 is one of the first transcripts to be found in the developing heart, where it precedes the expression of earliest cardiac differentiation markers (Charron and Nemer, 1999; Gilbert, 2006). Mutations in the *GATA4* gene have been found in patients with congenital heart defects (Garg *et al.*, 2003; Okubo *et al.*, 2004; Hirayama-Yamada *et al.*, 2005; Sarkozy *et al.*, 2005), but no mutations have been related to upper-limb malformations so far.

The NKX2.5 (MIM *600584) protein plays an important hole in the genesis of the heart. Mutations in the *NKX2.5* gene may lead to several types of cardiac malformations (Elliott *et al.*, 2003). Previous studies have shown that the GATA4 protein interacts with both the NKX2.5 (Durocher *et al.*, 1997) and the TBX5 (Garg *et al.*, 2003) protein, and that these interactions can lead to upregulation of cardiogenesis in cells under differentiation.

Because of the wide phenotypic variability found in the HOS patients, we screened for mutations in the *TBX5*, *GATA4* and *NKX2.5* genes in patients presenting with classical HOS, classical HOS with feet anomalies, patients with isolated upper-limb malformations, and patients with isolated heart defects. We selected 32 unrelated patients and classified them into four categories: typical HOS (n = 8), atypical HOS (n = 1), isolated hand anomalies (n = 7), and isolated heart defects (n = 16). Differential diagnosis for known conditions such as VACTERL sequence and Waardenburg syndrome was previously performed for exclusion from the sample. Informed consent in accordance with the guidelines established by the local institutional ethics boards was obtained.

Genomic DNA was isolated from all patients, their parents, and 50 healthy individuals, according to standard methods (Miller *et al.*, 1988). Primers used to analyze the *TBX5*, *GATA4*, and *NKX2.5* genes and the conditions for the polymerase chain reactions (PCR) were the same as previously described by other authors (Basson *et al.*, 1997; Garg *et al.*, 2003; Basson *et al.*, 1999, respectively). PCR products were submitted to direct sequencing, using an ABI Prism 3100 automatic sequencer according to manufacturer's protocol. The obtained sequences were compared with the wild-type *TBX5* (RefSeq NM_000192.3), *GATA4* (RefSeq NM_002052.3), or *NKX2.5* (RefSeq NM_004387.2) genomic sequences. The patients' clinical findings and detected mutations are listed in Table 1.

Four out of eight patients with typical HOS had mutations in the *TBX5* gene. Patient HH17, a woman with ASD and triphalangeal thumbs (Figure 1A), had a previously described pathogenic mutation (c.835C > T/ p.R279X) that does not affect the TBX5-binding domain, but causes the loss of its C-terminal portion (Li *et al.*, 1997), and is considered a hotspot mutation (Heinritz *et al.*, 2005b). The patient also carried a *TBX5* c.309C > T mutation, in heterozygosis. This is a polymorphic silent mutation according to the “The Human *TBX5* Gene Mutation Database” (Heinritz *et al.*, 2005a). The patient's son presented the same genotype, but a milder phenotype: shortened arms and no CHD.

Patient HH20 had a *de novo* novel mutation (c.374delG) in the *TBX5* gene that resulted in a premature stop codon. This mutation eliminated the binding domain of the TBX5 protein, giving rise to a truncated protein with only 148 amino acids. This patient had a very severe phenotype, with bilateral upper-limb malformations (bilateral humerus hypoplasia, bilateral absence of the radius, three metacarpal bones, and two phalangeal fingers on the right side; and three metacarpal bones, and three phalangeal fingers (two of them syndactylic) on the left side, besides a severe heart malformation characterized by ASD, a ventricular septal defect and pulmonary hypertension (Figure 1B). Another previously described mutation, which resulted in a premature stop codon at the same position, was associated with the occurrence of a severe upper-limb malformation, but no heart defects were mentioned for that patient (Yang *et al.*, 2000).

A novel mutation, c.678G > T/ p.K226N, located within the T-box domain of the *TBX5* gene, was found in patient HH24 and her father. Both of them presented with severe congenital heart disease (OS-ASD, atrioventricular septal defect, pulmonary stenosis, hypertension, and mitral insufficiency), besides upper-limb and other malformations (triphalangeal thumbs, 11 pairs of ribs, and shortened scapular bone; Figure 1C). This mutation was predicted to impair the function of the TBX5 protein (PSIC score difference 1.966; PolyPhen), leading to hydrophobicity and charge changes at buried site. It was not found in the 100 control chromosomes tested.

Patient HH25 had a c.804C > G mutation in the *TBX5* gene, a silent mutation that is not expected to be the cause of the typical HOS clinical features (ASD, VSD, mitral valve insufficiency, dilatation of the left atrium and the pulmonary artery, preaxial polydactyly of the right thumb with duplication of the distal phalanx (presence of nails was observed on both the duplicated phalanges), distal implantation of the left thumb, mild hemithorax hypoplasia, upper-limb supination limited to the right side, and a sacralized transition vertebra; Figure 1D). The mutation was not found in the 100 control chromosomes tested.

The atypical HOS patient HH8 had a *de novo* c.835C > T mutation, the same as found in Patient HH17. The clinical findings were ostium secundum atrial septal defect (OS-ASD) and upper-limb malformation (brachymesophalangism of the fifth finger and high implantation of the left thumb; Figure 1E), all of which are common findings in HOS (Li *et al.*, 1997). This patient also presented lower-limb malformation (agenesis of the distal phalanx of the fifth toe), which is an unusual finding among HOS patients. A recent study (Garavelli *et al.*, 2008) describes a family with three affected individuals, one of which presents a foot anomaly. The authors included this patient in the group of atypical Holt-Oram syndrome phenotypes. Faria *et al.* (2008) reported a *TBX5* missense mutation (V263M) in a Brazilian family with atrial septal defect and postaxial hexadactyly of both feet.

In four of our typical HOS patients, no mutations were detected. In addition to the possibility of the involvement of other genes, large deletions in the investigated genes might have gone undetected by direct sequencing (Borozdin *et al.*, 2006).

Two out of the 16 patients with isolated heart anomalies had mutations in *TBX5*, *GATA4*, and *NKX2.5*. Patient CHD5 carried the c.309C > T polymorphism in the *TBX5* gene. Her parents did not have this polymorphism. She had an isolated heart defect of the OS-ASD type, and her three brothers presented valve defects, but they were not available for mutation analysis. Patient CHD1 presented a presumptively pathogenic mutation in the *NKX2.5* gene (c.73C > T/p.R25C), together with the c.543C > T polymorphism in the *GATA4* gene. This patient had OS-ASD, but his father who carried the same mutations showed no heart malformation. The *NKX2.5* p.R25C mutation was previously described in patients with a wide spectrum of congenital heart diseases, from conduction defects to Tetralogy of Fallot (Benson *et al.*, 1999; McElhinney *et al.*, 2003). However, the frequency of this mutation in black control subjects was 4.7% (Goldmuntz *et al.*, 2001), which is suggestive of low penetrance or no involvement in CHD.

Two out of the seven patients with isolated upper-limb anomalies had mutations in *TBX5* and/or *GATA4*. Patient HH2 had radioulnar synostosis of the left arm and hypoplasia of the distal and middle phalanges of the first and fifth digits (Figure 1F). At right, she had synostosis of ulna and humerus, a hypoplastic radius, and only two digital rays of the right hand (the first-finger phalanges were hypoplastic, and only two metacarpal bones were observed), as shown in Figure F. No congenital heart disease was diagnosed in the patient or in her family (ECO and ECG were normal). Mutational analysis of the *GATA4* gene showed two novel *de novo* intron mutations, c.[997+23A > T; 997+56C > A], in the same allele. These mutations were located far from the splicing sites, and the potential instability of the mRNA remains to be accessed. They were not found in the 100 control chromosomes tested. The patient also presented the *TBX5* c.309C > T polymorphism in homozygosis.

Patient HH4 had a hypoplastic thumb with low implantation and shortened phalanges of the right hand. The left hand showed radial deviation and absence of the thumb (Figure 1G). No congenital heart disease was observed in him or his parents (ECO and ECG were normal). The *GATA4* gene showed a novel nonsense mutation, c.392C > G (p.A113G), located in the transcriptional activation domain 1. This variant was predicted to be benign (PSIC score difference 1.191), according to the PolyPhen program (Ramensky *et al.*, 2002). It was not found in the patient's parents or in the 100 control chromosomes.

So far, *GATA4* mutations have not been described in association with isolated upper-limb defects, but this is a possibility that deserves further investigation.

**Figure 1:**
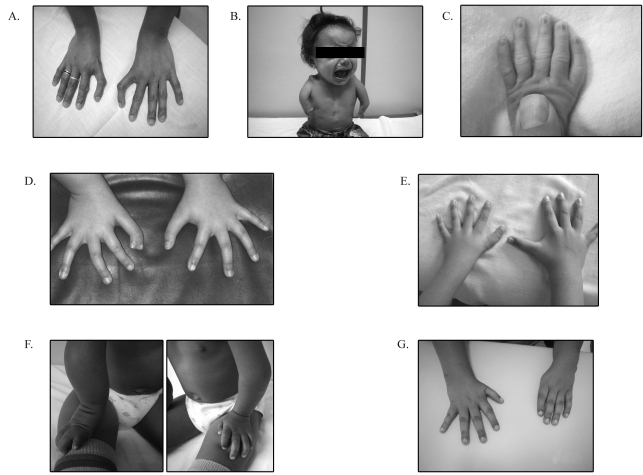
Upper-limb malformations found in patients. (A) HH17: triphalangeal thumbs. (B) HH20: at right, bilateral humerus hypoplasia and absence of radius, three metacarpal bones, and two phalangeal fingers; at left, three metacarpal bones, and three phalangeal fingers, two of them syndactylic. (C) HH24: triphalangeal thumbs. (D) HH25: preaxial polydactilia of the right thumb with duplication of the distal phalanx (presence of nails was observed on both the duplicated phalanges), distal implantation of the left thumb. (E) HH8: brachymesophalangism of the fifth finger and high implantation of the left thumb. (F) HH2: At left, radioulnar synostosis, and hypoplasia of the distal and middle phalanges of the first and fifth digits; at right, synostosis of ulna and humerus, hypoplastic radius, two digital rays of hand. (G) HH4: At right, hypoplastic thumb (low articulated) and short phalanges; at left, radial deviation, and absence of the thumb.

## Figures and Tables

**Table 1:** Clinical findings in patients with mutations

Patient	Gender	Phenotype					Mutation		
		Typical HOS	Atypical HOS	Only hand defects	Only heart defects		Gene	Exon	Genotype
HH17	F	Fam	-	-	-		*TBX5*	4	c.309C > T (SNP)
								8	c.835C > T (p.R279X)
HH20	F	Sp	-	-	-		*TBX5*	5	**c.374delG (p.G125fsX149)**
HH24	F	Fam	-	-	-		*TBX5*	7	**c.678G > T (p.K226N)**
HH25	F	Sp	-	-	-		*TBX5*	8	**c.804C > G (SNP)**
HH8	M	-	Sp*	-	-		*TBX5*	8	c.835C > T (p.R279X)
HH2	F	-	-	Sp	-		*TBX5*	4	c.309C > T (SNP)**
							*GATA4*	4	**c.[997+23A > T; 997+56C > A]**
HH4	M	-	-	Sp	-		*GATA4*	1	**c.392C > G (p.A131G)**
CHD1	M	-	-	-	Sp		*NKX2.5*	1	c.73C > T (p.R25C)
							*GATA4*	1	**c543C > T (SNP)**
CHD5	F	-	-	-	Fam		*TBX5*	4	c.309C > T (SNP)

HOS: Holt-Oram syndrome. HH: heart-hand syndrome. CHD: congenital heart defect. Sp: sporadic cases. Fam: familial cases. **Bold:** novel mutations and polymorphisms. *Feet anomaly. **Mutation found in homozygosis.
